# Antimicrobial prophylaxis for colorectal surgery

**DOI:** 10.1590/S1516-31802012000300012

**Published:** 2012-07-12

**Authors:** Richard L.Nelson, Anne Marie Glenny, Fujian Song

## Abstract

**BACKGROUND:**

Research shows that administration of prophylactic antibiotics before colorectal surgery prevents postoperative surgical wound infection (SWI). The best antibiotic choice, timing of administration and route of administration remain undetermined.

**OBJECTIVES:**

To establish the effectiveness of antimicrobial prophylaxis for the prevention of SWI in patients undergoing colorectal surgery: specifically to determine,

1 Whether it reduces risk of SWI.

2 The target spectrum/a of bacteria (aerobic and/or anaerobic).

3 The best timing and duration of antibiotic administration.

4 The most effective route of antibiotic administration (intravenous, oral or both).

5 Whether any antibiotic is clearly more effective than the currently recommended gold standard.

**CRITERIA FOR CONSIDERING STUDIES FOR THIS REVIEW:**

Central, Medline, and Embase, were searched from January, 1980 to December, 2007.

**SELECTION CRITERIA:**

Randomized controlled trials of prophylactic antibiotic use in elective and emergency colorectal surgery, with SWI as an outcome.

**DATA COLLECTION AND ANALYSIS:**

Data were abstracted and reviewed by three authors for only the single, dichotomous outcome of SWI.

**MAIN RESULTS:**

The review included 182 trials (30,880 participants), and 50 different antibiotics, including 17 cephalosporins. Many studies had multiple variables that separated the two study groups and could not be compared to other studies that tested one antibiotic and had a single variable separating the two groups. Meta-analyses demonstrated a statistically significant difference in postoperative SWI when prophylactic antibiotics were compared to placebo/no treatment [relative risk (RR) 0.30, 95% confidence intervals (CI) 0.22 to 0.41}. No statistically significant differences were shown when comparing short- and long-term duration of prophylaxis (RR 1.06, 95% CI 0.89 to 1.27); or single dose versus multiple dose antibiotics (RR 1.17, 95% CI 0.67 to 2.05). Additional aerobic coverage and additional anaerobic coverage both showed statistically significant improvements in SWI rates (RR 0.41, 95% CI 0.23 to 0.71 and RR 0.55, 95% CI 0.35 to 0.85, respectively); as did combined oral and intravenous antibiotic prophylaxis when compared to intravenous alone (RR 0.55, 95% CI 0.41 to 0.74), or oral alone (RR 0.34, 95% CI 0.13 to 0.87). Established gold standard regimens were no less effective than any other antibiotic choice.

**AUTHORS’ CONCLUSIONS:**

Antibiotics covering aerobic and aerobic bacteria should be delivered orally and intravenously prior to colorectal surgery. Antibiotics delivered within this framework will reduce the risk of postoperative SWI by at least 75%. Further research is required to establish the optimal timing and duration of dosing, and frequency of longer-term adverse effects such as Clostridium difficile pseudomembranous colitis.

This is the abstract of a Cochrane Review published in the Cochrane Database of Systematic Reviews (CDSR) 2009, Issue 1, Art. No. CD001181, DOI: 10.1002/14651858.CD001181.pub3 (http://onlinelibrary.wiley.com/doi/10.1002/14651858.CD001181.pub3/abstract). For full citation and authors details see reference 1.

For Latin America and the Caribbean, the full text is freely available from: http://cochrane.bvsalud.org/cochrane/show.php?db=reviews&mfn=602&id=CD001181&lang=pt&dblang=&lib=COC&print=yes.
